# Using citizen science data for predicting the timing of ecological phenomena across regions

**DOI:** 10.1093/biosci/biae041

**Published:** 2024-07-09

**Authors:** César Capinha, Ana Ceia-Hasse, Sergio de-Miguel, Carlos Vila-Viçosa, Miguel Porto, Ivan Jarić, Patricia Tiago, Néstor Fernández, Jose Valdez, Ian McCallum, Henrique Miguel Pereira

**Affiliations:** Centre of Geographical Studies, Institute of Geography and Spatial Planning of the University of Lisbon, Lisbon, Portugal; Associate Laboratory Terra Lisbon, Portugal; BIOPOLIS, CIBIO, InBIO Associate Laboratory, University of Porto, Porto, Portugal; University of Lisbon, LisbonPortugal; Department of Agricultural and Forest Sciences and Engineering, University of Lleida, Lleida, Spain; Forest Science and Technology Centre of Catalonia, Solsona, Spain; BIOPOLIS, CIBIO, InBIO Associate Laboratory; Museu de História Natural e da Ciência, University of Porto, Porto, Portugal; BIOPOLIS, CIBIO, InBIO Associate Laboratory, , University of Porto, Porto; University of Lisbon, Lisbon; Mértola Biological Station, Mértola, Portugal; Université Paris-Saclay, CNRS, AgroParisTech, Ecologie Systématique Evolution Paris, France; Biology Centre of the Czech Academy of Sciences, Institute of Hydrobiology, České Budějovice, Czech Republic; Centre for Ecology, Evolution, and Environmental Changes & CHANGE--Global Change and Sustainability Institute, at Faculty of Sciences, University of Lisbon, Lisbon, Portugal; German Centre for Integrative Biodiversity Research (iDiv) Halle-Jena-Leipzig, Leipzig, Germany; Institute of Biology from the Martin Luther University Halle-Wittenberg, Halle, Germany; German Centre for Integrative Biodiversity Research (iDiv) Halle-Jena-Leipzig, Leipzig, Germany; Institute of Biology from the Martin Luther University Halle-Wittenberg, Halle, Germany; International Institute for Applied Systems Analysis, Laxenburg, Austria; German Centre for Integrative Biodiversity Research (iDiv) Halle-Jena-Leipzig, Leipzig, Germany; Institute of Biology from the Martin Luther University Halle-Wittenberg, Halle, Germany; BIOPOLIS and CIBIO, Porto, Portugal

**Keywords:** citizen science, digital data, ecological monitoring, phenological niche, seasonality prediction

## Abstract

The scarcity of long-term observational data has limited the use of statistical or machine-learning techniques for predicting intraannual ecological variation. However, time-stamped citizen-science observation records, supported by media data such as photographs, are increasingly available. In the present article, we present a novel framework based on the concept of relative phenological niche, using machine-learning algorithms to model observation records as a temporal sample of environmental conditions in which the represented ecological phenomenon occurs. Our approach accurately predicts the temporal dynamics of ecological events across large geographical scales and is robust to temporal bias in recording effort. These results highlight the vast potential of citizen-science observation data to predict ecological phenomena across space, including in near real time. The framework is also easily applicable for ecologists and practitioners already using machine-learning and statistics-based predictive approaches.

Ecological phenomena with intraannual variation, such as species phenology, migrations, behavior, or productivity levels, are key drivers and indicators of the structure, status, and functioning of ecological systems (Tang et al. 2016). Spatial predictions of such phenomena over short and long time frames now serve a variety of important fundamental and applied purposes, including improved understanding of ecological processes (Houlahan et al. 2017, Dietze et al. 2018), anticipation of ecological risks (e.g., Kim et al. 2023), management of threats to biodiversity (e.g., Henden et al. 2022, Slingsby et al. 2023), and the promotion of sustainable use of natural resources (e.g., Marolla et al. 2021). These contributions are of growing significance, given escalating environmental changes and mounting human pressures on biodiversity (Pereira et al. 2012).

Statistical or machine-learning-based predictions of ecological phenomena that change over time rely on algorithm-based identification of predictive features, either in the temporal progression of the event itself or in putative environmental drivers. Although this approach is generally straightforward, its use is dependent on the availability of observational data amenable to model fitting. More specifically, commonly used data-driven modeling techniques, such as state–space models or custom-built machine-learning architectures are mostly fitted using time series of the phenomenon of interest, preferably collected over representative geographical extents (e.g., Rammer and Seidl 2019, Marolla et al. 2021, Morera et al. 2021, Lofton et al. 2022). Unfortunately, data sets meeting these requirements are often nonexistent or remain temporally or spatially limited for many ecological phenomena.

At the same time, the number of citizen-science biodiversity observation records in public repositories, such as eBird (ebird.org), the Global Biodiversity Information Facility (GBIF; gbif.org), Observation (observation.org) or iNaturalist (inaturalist.org), has been rising steeply (Bonney 2021, Callaghan et al. 2023). These data are frequently georeferenced with high precision, time stamped, and accompanied by visual media, including photographs (Groom et al. 2021, Meeus et al. 2023). As such, they represent a potentially valuable source of information on the spatiotemporal dynamics of ecological phenomena. Previous research has already demonstrated their usefulness for temporal ecology research, such as in measuring flight periods for Lepidoptera species (Belitz et al. 2023) or in estimating the flowering period of plant species (Puchałka et al. 2022). However, despite their widespread availability, the use of presence-only, predominantly opportunistic data for temporal modeling is challenging because of the lack of temporal replicability, temporal recording biases, and uneven spatial coverage. To minimize these limitations, previous researchers have selected records from areas with temporal replicability (e.g., where multiple records are available within the same year) from which temporal trends are then interpolated (e.g., Belitz et al. 2020, Puchałka et al. 2022). Although practical and seemingly effective (Pearse et al. 2017, Belitz et al. 2020), this approach disregards potentially informative records in regions with low or null temporal replicability. Moreover, the need for temporal replicability also creates data availability issues, similar to those of time-series data, limiting the phenomena and regions that can be modeled.

Box 1. **Conceptual framework.**

This conceptual framework describes the ecological components represented in our modeling approach. We base the conceptual framework on the concept of the relative phenological niche, which refers to the timing of phenological events as a function of temporal variation in biotic and abiotic factors—that is, relative to environmental drivers (Post 2019). Relative phenological niches form the set of temporal environmental conditions within which a phenological event occurs, a concept that can be represented as an *n*-dimensional hypervolume (figure 1a). For example, if a hedgehog is active on a specific day of the year, this means that the conditions for relevant biotic (e.g., food resources) and abiotic (e.g., temperature and precipitation) factors—with reference to the specific day and place of the observation—are within the boundaries of the hypervolume of hedgehog activity. Relevantly, these hypervolumes can be empirically sampled using records of the observation of the event, where each record represents a point in its *n*-dimensional space (figure 1b). The comprehensiveness of the sampling inherently depends on the representativeness of the set of observation records. However, with many phenomena now being represented by thousands or tens of thousands of opportunistic records (Bonney 2021, Klinger et al. 2023) it seems plausible to assume that a good representativeness can often be achieved.

**Figure 1.**
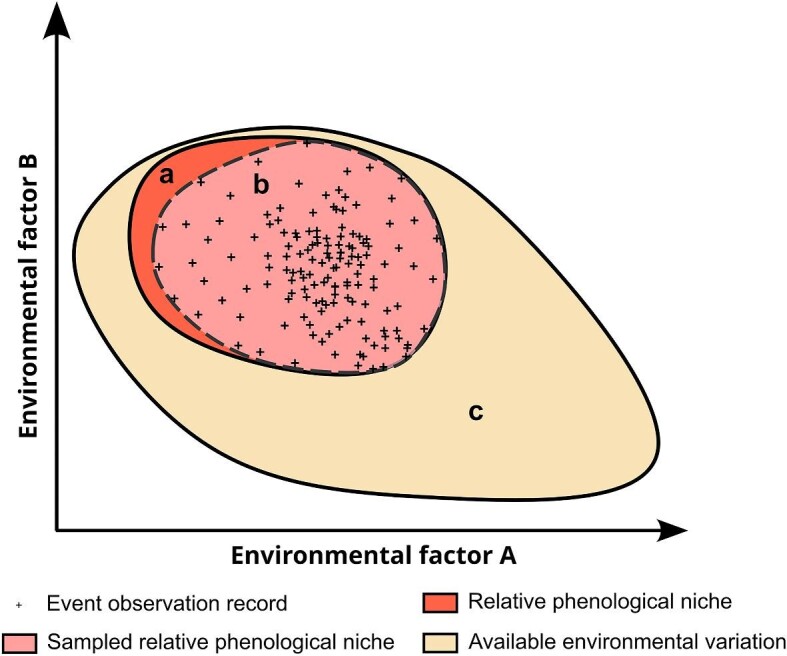
Schematic representation of the conceptual framework underlying the modeling approach. Hypervolumes of temporal environmental conditions are represented describing the relationship between the relative phenological niche of a hypothetical phenomenon of interest (a), a subset of this niche that is represented by available presence-only observation records (b), and the full set of temporal environmental variation that is available in locations where the phenomenon occurs (c). Delineation of hypervolumes is made along a simplified two-dimensional space defined by the timing of two hypothetical environmental drivers.

We also consider the assembly of a set of environmental conditions to contrast with those representing the relative phenological niche. For that purpose, we use the set of temporal environmental conditions that are available in places where the phenomenon occurs—that is, the so-called realized environmental conditions (figure 1c; Post 2019).

These conditions are sampled using records with the same geographical coordinates as observation records (guaranteeing that the sampling is made in areas where the event occurs) but with dates randomly selected from the temporal span of the event records. Hereafter, we call these records *temporal pseudoabsences*, because they are conceptually similar to pseudoabsence records used in species distribution modeling for sampling the geographical space available (Phillips et al. 2009).

In summary, our framework assembles a data set representing the temporal environmental conditions associated with observation of the phenomenon of interest and with the full set of conditions in places where the phenomenon occurs. Discriminative algorithms are then used to distinguish between these two sets, and the predictions can be interpreted as the probability of the represented conditions belonging to the relative phenological niche of the represented phenomenon.

In this study, we propose a novel, data-driven approach for predicting the intraannual timing of occurrence of ecological phenomena using opportunistic presence-only records. The approach is grounded in ecological theory (box 1) and assumes that any observation record of the phenomenon of interest reflects the temporal match of physical and biological conditions suitable for its occurrence. By jointly sampling the set of conditions represented across multiple records, our approach constructs a representation of the temporal environmental space under which the phenomenon occurs—that is, its phenological niche (Post 2019). This approach can integrate all occurrence data available and is not reliant on regional temporal replicability. To demonstrate its effectiveness, we use it to provide daily predictions of the occurrence of adult invasive Japanese beetles (*Popillia japonica*) and fruiting bodies of the winter chanterelle mushroom (*Craterellus tubaeformis*) across Europe and North America. We also show its applicability for management-related tasks by using environmental predictors enabling the near real time prediction of these two ecological events. Our approach is conceptually intuitive and straightforward to implement for ecologists experienced with machine-learning or statistics-based predictive modeling. It also provides a promising research avenue to harness the vast and growing amounts of citizen-science biodiversity observation data for predicting the timing of ecological phenomena.

## A framework for predicting the timing of ecological phenomena using citizen-science data

To demonstrate the implementation of the methodological framework derived from the conceptual framework (box 1), we use phenological events related to the Japanese beetle and the winter chanterelle mushroom. The Japanese beetle is a highly problematic invasive species known to feed on hundreds of plant species, causing significant economic losses (Potter and Held 2002). This beetle is well established in North America and the Azores and has recently established in northern Italy, raising concerns of a rapid invasion of Europe from there (EFSA 2019). Early detection surveys can be effective in preventing the spread of this species, and the adult life stage is particularly suitable for such surveys (EFSA 2019). Therefore, it is essential to understand when adults of this species are likely to be observed, especially in areas where their presence is uncertain, to determine the appropriate timing for implementing surveillance efforts.

The winter chanterelle is a popular edible mushroom found in Europe and North America. In some areas of these regions, the harvesting of wild edible mushrooms is regulated to avoid excessive human pressure on areas of occurrence (Copena et al. 2022). Importantly, the timing and abundance of mushroom fruiting bodies determine the level of human pressures (Górriz-Mifsud et al. 2017); therefore, having prior knowledge about the timing of their occurrence can aid in management decisions. In addition, it can assist the collectors in planning their harvest activities, being of potential benefit to a large community of people.

**Figure 2.**
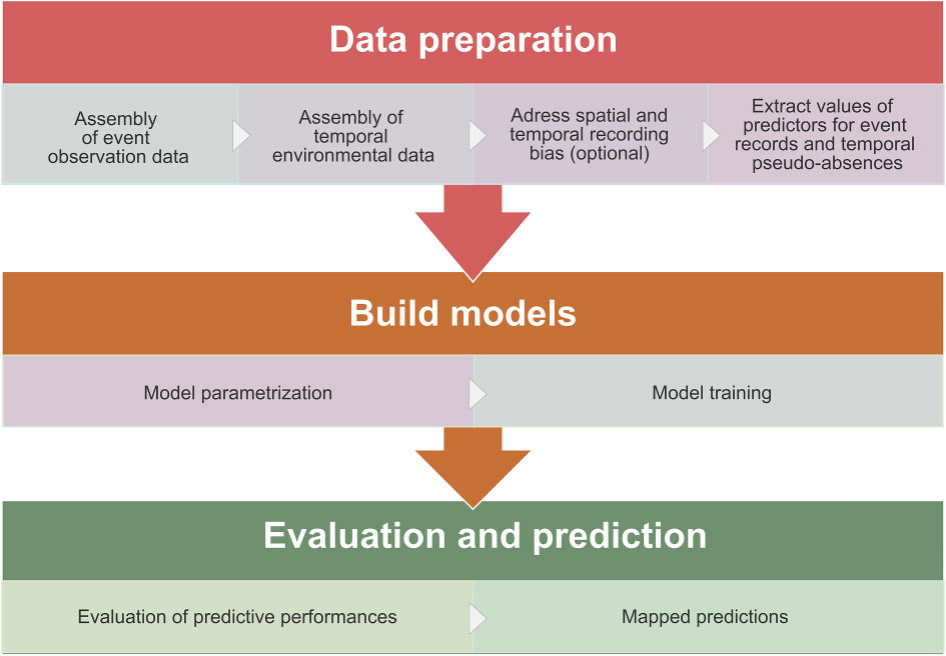
Schematic diagram representing the main components and steps of the methodological framework.

To provide a clearer understanding of the methodology described below, we outline the main steps of our framework in figure 2.

### Step 1: Assembly of event observation data

We obtained observation records for both species from the GBIF (www.gbif.org), which is a leading aggregator of biodiversity observation records, including those from citizen science platforms such as iNaturalist (www.inaturalist.org). For the winter chanterelle, we also included records from Mushroom Observer (https://mushroomobserver.org), another citizen science platform not included in GBIF. We limited our data set to records with photographic evidence, full date of observation (i.e., day, month, and year), and geographic coordinates with a spatial precision greater than 0.1 decimal degrees (approximately 4–11 kilometers [km], depending on latitude). We included records from 2015 to 2021 and, to ensure data quality, we removed GBIF records where the observation date was the first day of the month and the observation time was 00:00:00. These records generally only provide the month and year and are assigned the first day of the month by default (Belitz et al. 2023). We then assessed the photographic evidence supporting each remaining record. For the Japanese beetle, we retained only records where the photograph showed an adult life stage and no signs of the specimen being dead. For the winter chanterelle, we kept records supported by photographs of fruiting bodies that showed no signs of significant deterioration.

### Step 2: Environmental data

The timing of ecological events can be influenced by a multitude of biotic and abiotic factors. However, for the two phenomena being modeled, weather-related factors are believed to be the main drivers of their seasonality, as has been evidenced by previous studies (Diez et al. 2013, EFSA 2019). Therefore, we used daily spatial time series of minimum temperature, mean temperature, maximum temperature, total precipitation, snow depth, and wind speed to capture the environmental conditions associated with the occurrence of these events.

We sourced these data from the AgERA5 data set (Boogaard et al. 2020), which provides daily weather maps at a spatial resolution of 0.1 degree. The data was collected for the period from 2014 to 2021, but its availability has a delay of approximately one month after the last day represented. Therefore, to exemplify the implementation of the framework using a source that provides up to date weather data, we collected the same set of variables from the Global Forecast System (GFS), a weather forecast model from the National Centers for Environmental Prediction (https://www.ncei.noaa.gov/). The GFS provides forecasts of weather conditions at intervals of up to 3 hours and runs four times a day at 00:00, 06:00, 12:00, and 18:00 UTC. To ensure consistency between the two data sources, we extracted the forecasted conditions for the first 6 hours of each model run and aggregated them to a daily resolution. We also resampled AgERA5 data to 0.25-degree cell size (approximately 28 km at the equator), the spatial resolution of GFS data. The processing of spatial weather data was performed in R (R Core Team 2022) using functions provided by the “terra” and “raster” packages (Hijmans et al. 2023).

### Step 3: Addressing spatial and temporal recording bias (optional)

Biodiversity observation data are often geographically and temporally biased—particularly, opportunistically collected records (Isaac and Pocock 2015). To minimize these biases in our models, we applied a set of procedures described next. We note, however, that this step is optional within our framework and may be omitted if there are reasons to expect that the data is not significantly affected by recording biases.

To address spatial bias, such as disproportionately high numbers of records in some regions, potentially dominating the overall patterns in the data (i.e., geographic overrepresentation), we first randomly selected only one record per each combination of day and 0.25 × 0.25-degree grid cell, which is the resolution of our environmental data. We also accounted for overrepresentation at the regional scale by creating a regular grid of 250 × 250 km squares covering the entire study area and counting the number of records in each square. We identified squares that exceeded the upper outlier threshold (Q3 + 1.5 × IQR, where Q3 is the upper 25% quantile, and IQR = Q3, the lower 25% quantile) and randomly selected a number of observations equal to the threshold value (i.e., Q3 + 1.5 × IQR) to address overrepresentation.

To address temporal biases in data, we used *Pinus* spp. as a benchmark taxonomic group, which we expect to experience variability in record availability mainly because of variation in observation effort rather than changes in the taxa phenology itself (see the supplemental material for an expanded rationale). From GBIF, we downloaded and cleaned the *Pinus* spp. observation records made in the northern hemisphere between 2015 and 2021 (i.e., the geographical and temporal range of the event observation data; see the supplemental material). We then generated an equal number of records having the same coordinates but with randomly generated dates within the same temporal range. We extracted calendar and weather predictors for each record (day of the week, month, average temperature of the day, total precipitation of the day, and average wind speed of the day) and used a GLM with a binomial error distribution to relate the two classes of records (see the supplemental material). The model is assumed to capture well the propensity for having more citizen science records simply because conditions are more favorable to observers (i.e., preferred days of the week, months, and weather conditions; see the “Model predictions” section). Subsequently, we applied the model to estimate levels of sampling effort for the Japanese beetle and winter chanterelle data sets based on the same predictors. Inverse probability weighting was used to correct for temporal bias in these data sets, where the probability of each observation being used in subsequent modeling steps was inversely proportional to the level of observation effort predicted. Specifically, we built a second data set of observation records for each event, where the probability of each original observation being included was inversely proportional to the level of observation effort predicted. For a more detailed explanation, please refer to the supplemental material.

Although we expect this procedure to minimize temporal biases in the data, we also acknowledge that it still has limitations such as, for instance, the omission of additional drivers of observation effort (e.g., national holidays). Bearing this in mind, all subsequent analyses were carried out using both the temporally corrected observation data and the data without this correction.

### Step 4: Extract values of predictors for event records and temporal pseudoabsences

To represent the environmental conditions at the time of each observation record, we calculated a comprehensive set of 67 features (listed in supplemental table S1). These features represent the geographical coordinates of each record of the event and yearlong to subweekly conditions in mean, maximum, and minimum temperature, accumulated precipitation, wind speed, and snow depth. Importantly, each feature value was calculated in reference to the date of the record, meaning that they capture environmental conditions observed in the preceding periods—for example, days, weeks, months, year (table S1).

As was mentioned in the conceptual framework section (box 1), we also assembled a set of environmental conditions to contrast with those representing relative phenological niches, enabling the use of discriminative modeling algorithms. To this end, we generated a set of temporal pseudoabsences by generating, for each observation record, a set of 12 records having the same geographical coordinates but dates drawn at random from the temporal range of the event observation data (i.e., from 2015 to 2021). The use of 12 temporal pseudoabsences per event record was determined empirically on the basis of preliminary tests evaluating the time taken for model training and internal cross-validation values. Although this ratio allowed us to achieve good overall predictions (see the “Model predictions” section), we acknowledge that future work could investigate this further and additional optimization may be possible. For each temporal pseudoabsence record, we extracted the same set of 67 features used to characterize event records, providing a representation of the environmental conditions available over time in locations where the species occur (figure 1).

### Step 5: Model training

To differentiate between the conditions associated with the timing of observation of events and the full range of conditions available, we employed Random Forests (RF) and Boosted Regression Trees (BRT), two well-performing machine-learning algorithms commonly used in ecological research (Cutler et al. 2007, Elith et al. 2008). Although we opted for these algorithms, it is important to note that many other statistical or machine-learning techniques could be similarly employed (e.g., see Norberg et al. 2019 for alternatives). For RF, we used the “randomForest” function of the R package with the same name (Liaw and Wiener^ 2002^), specifying a total of 2000 individual trees and remaining parameters set at default values (but see below for an exception regarding “sampsize”). For BRT, we used the “gbm.step” function of the “dismo” package (Hijmans et al. 2017), setting a tree complexity of 3, a learning rate of 0.005, four internal cross-validation folds, a bag fraction of 66%, and a maximum of 7500 individual trees.

Given the high class imbalance in our data sets (i.e., 12 temporal pseudoabsences per event record), we took steps to prevent model fitting problems such as overclassification of the majority class (Valavi et al. 2022). For RF training, we used the event observation records and an equal number of randomly selected temporal pseudoabsences for fitting each tree, as is allowed by the “sampsize” parameter. In BRT, we assigned a relative weight of 1:12 (i.e., 8.3%) to each temporal pseudoabsence, as is allowed by the “site.weights” parameter. Before each model training event, we also measured the Pearson correlation coefficient among environmental variables, retaining only the minimum set of predictors with an absolute correlation value lower than .8 (Valavi et al. 2022) using the “findCorrelation” function from the “caret” package (Kuhn 2008) for R.

### Step 6: Evaluation and validation of predictive performances

To evaluate the predictive performance of models, we first measured their capacity to correctly classify event observation records and pseudoabsences. This was evaluated for each year independently, where model training used data for the remaining years (i.e., temporally independent data). We used the area under the receiver operating characteristic curve (area under the curve, AUC) to measure the agreement between predictions and the actual record (i.e., observation or temporal pseudoabsence). In the context of this work, the AUC measures the probability that observation events receive higher probability values than records generated randomly over time. AUC values range from 0 to 1, where a score of .7 or above is considered an acceptable level of discrimination (e.g., Valavi et al. 2022).

Although the above-described evaluation procedures assess the performance of each model, they do not allow for a comparison between models using temporally corrected observation data and models using uncorrected data. To allow such comparisons, we performed a second set of evaluations comparing predictions with raw observation data (i.e., without spatial or temporal correction) for regions left out of model training and where higher data reliability can be expected. Specifically, for the adult stage of the Japanese beetle, we performed this validation using observation data from northern Italy (*n* = 214), a region of high relevance for invasion surveillance of the species in Europe and where the species has received significant attention in recent years (EFSA 2019). For the fruiting bodies of the winter chanterelle, we used data from Denmark, the country with the highest number of observation records (*n* = 460) and where the species is foraged and marketed (Gry and Andersson 2014).

To perform this assessment, we predicted the average probability of observing the event across the study area on each day, for years with 10 or more observation records in validation regions (i.e. 2019–2021 for adults of the Japanese beetle and 2017–2021 for the fruiting bodies of the winter chanterelle). To measure the association between the timing of observation of events and model predictions, we calculated the point-biserial correlation between the average predicted probability in the region and the presence (coded as 1) or absence (coded as 0) of event observations using 10-day time steps. For the two evaluation procedures (i.e., temporally corrected versus uncorrected data), we measured the performance of BRT, RF, and of an ensemble model, which is the average of the predictions of the two former algorithms.

### Step 7: Mapped predictions

To showcase the potential of our framework and assess the spatial patterns of temporal change in our predictions, we generated daily prediction maps for both events (i.e., adult stage of the Japanese beetle and fruiting bodies of the winter chanterelle) in Europe for the year 2021. The predictions were produced using the ensemble model trained with spatially and temporally corrected observations and AgERA5 predictor data. To avoid extrapolating beyond sampled environmental conditions, we masked all regions deemed unsuitable for the Japanese beetle (cf. EFSA 2019) and regions outside the distribution range of the winter chanterelle, as is represented by its observation data. To enhance visualization, we used bilinear interpolation to downsample predictions to a resolution of 0.02 degrees (approximately 2 km) using the “resample” function provided by the “raster” package (Hijmans et al. 2023).

## Model predictions

In total, we obtained 15,529 observation records of adult Japanese beetles and 3057 of fruiting bodies of the winter chanterelle, of which 10,308 and 1726 were kept for model training (respectively) after accounting for geographic overrepresentation (supplemental figures S2 and S3). For both events, the number of records increased substantially over time, with 2020 and 2021 holding more records than the previous 5 years (2015 to 2019) combined (figures S2a and S3a). Event records for the Japanese beetle were distributed in regions of Asia, Europe, Central America, and North America, but with the vast majority concentrated in the latter, more specifically in the United States (figure S2b–S2d). For the winter chanterelle, event records were almost entirely distributed in Europe and North America, except for a few records in Central America and Japan (figure S3b–S3d).

### Temporal bias correction

The GLM model used to examine the relationships between the availability of records for the benchmark taxa (*Pinus* spp.) with calendar- and weather-related variables yielded convincing results. The model revealed a significant (*α* = .05) positive relationship between record availability and warmer days, low precipitation, and low wind intensity (supplemental table S2). It also identified a significantly higher propensity for observation records to be made during the weekend, and in May, July, and August, in comparison to Friday and April (day of the week and month used as reference level, respectively). Conversely, significantly lower numbers of records were identified for all remaining months except June (i.e., January, February, March, September, October, November, and December), as well as for Mondays and Tuesdays.

### Predictive performances and mapped predictions

The BRT and RF algorithms, along with the ensemble model, consistently demonstrated very good predictive performance when evaluated on years that were not included in the model training, achieving an AUC of .81 or higher (tables 1a and 1b). The models predicting the timing of occurrence of adult Japanese beetles exhibited a higher discrimination capacity (average AUC = .9, standard deviation [SD]  = 0.1) than those for the fruiting bodies of the winter chanterelle (average AUC = .84, SD = 0.2). Models trained on temporally corrected and uncorrected data demonstrated similar levels of accuracy overall. The residual values displayed significant variation across the study areas (supplemental figures S4–S7), indicating that classification errors were not spatially clustered, and the global AUC values were geographically representative.

**Table 1a.** Values of area under the curve (AUC) for models predicting the timing of occurrence of adult Japanese beetles (*Popillia japonica*) across the whole study areas, from 2015 to 2021.

Model	Boosted regression trees				Random forests				Ensemble			
Weather data	AgERA5		GFS		AgERA5		GFS		AgERA5		GFS	
Temporal correction	Yes	No	Yes	No	Yes	No	Yes	No	Yes	No	Yes	No
2015	0.91	0.93	NA	NA	0.9	0.92	NA	NA	0.91	0.93	NA	NA
2016	0.9	0.89	0.9	0.88	0.89	0.89	0.88	0.88	0.9	0.89	0.89	0.88
2017	0.9	0.91	0.9	0.9	0.9	0.91	0.9	0.9	0.9	0.91	0.9	0.91
2018	0.9	0.91	0.89	0.9	0.9	0.9	0.9	0.9	0.9	0.91	0.9	0.9
2019	0.9	0.91	0.9	0.91	0.9	0.91	0.9	0.91	0.9	0.91	0.9	0.91
2020	0.91	0.91	0.9	0.91	0.9	0.91	0.9	0.9	0.9	0.91	0.9	0.91
2021	0.9	0.91	0.9	0.9	0.9	0.9	0.9	0.9	0.9	0.91	0.9	0.9

*Note:* Predictions are compared with data for years that were excluded from model training. The AUC values are shown for models trained with observation data corrected for temporal and spatial bias, and for spatial bias only, for models using AgERA5 weather data and Global Forecast System data (GFS).

**Table 1b.** Values of area under the curve (AUC) for models predicting the timing of occurrence of fruiting bodies of the winter chanterelle (*Craterellus tubaeformis*) across the whole study areas, from 2015 to 2021.

Model	Boosted regression trees				Random forests				Ensemble			
Weather data	AgERA5		GFS		AgERA5		GFS		AgERA5		GFS	
Temporal correction	Yes	No	Yes	No	Yes	No	Yes	No	Yes	No	Yes	No
2015	0.83	0.81	NA	NA	0.84	0.83	NA	NA	0.84	0.82	NA	NA
2016	0.84	0.85	0.83	0.82	0.85	0.85	0.84	0.83	0.85	0.86	0.84	0.83
2017	0.86	0.87	0.85	0.86	0.88	0.88	0.87	0.86	0.87	0.87	0.86	0.86
2018	0.84	0.85	0.85	0.86	0.85	0.87	0.85	0.86	0.85	0.87	0.85	0.86
2019	0.84	0.84	0.85	0.85	0.86	0.85	0.86	0.85	0.85	0.85	0.86	0.85
2020	0.83	0.83	0.82	0.82	0.84	0.84	0.83	0.83	0.84	0.83	0.83	0.83
2021	0.83	0.83	0.81	0.83	0.84	0.84	0.83	0.84	0.84	0.84	0.83	0.84

*Note:* Predictions are compared with data for years that were excluded from model training. The AUC values are shown for models trained with observation data corrected for temporal and spatial bias, and for spatial bias only, for models using AgERA5 weather data and Global Forecast System data (GFS).

**Figure 3.**
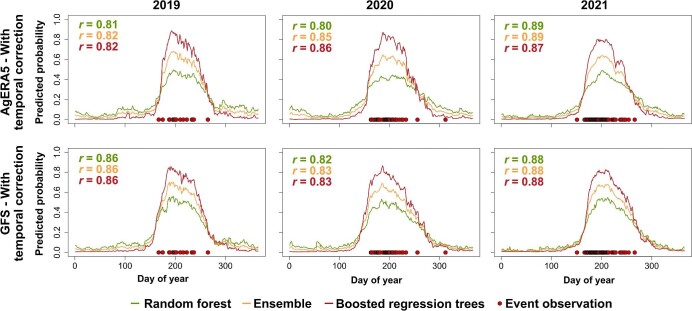
Continuous predictions of the timing of occurrence of adult Japanese beetles (*Popillia japonica*) in northwest Italy from 2019 to 2021. Predictions are shown for models trained with observation data corrected for temporal and spatial bias, for models using AgERA5 weather data and Global Forecast System data. Values of point biserial correlation coefficient (*r*) are provided, measuring the association between predicted values and the dates of actual observations. All values are statistically significant (*α* = .001).

**Figure 4.**
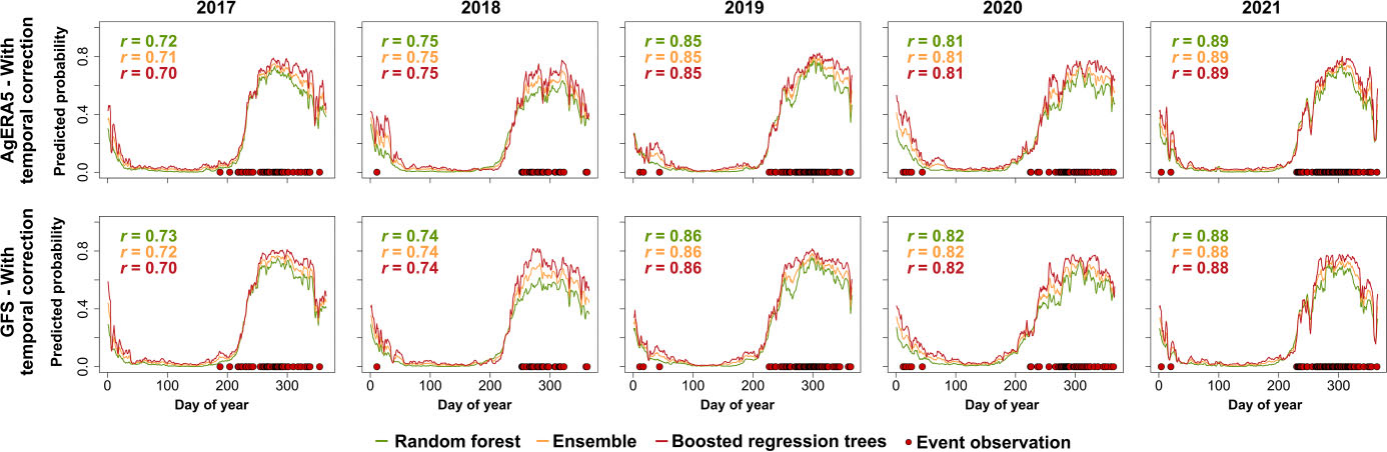
Continuous predictions of the timing of occurrence of fruiting bodies of the winter chanterelle (*Craterellus tubaeformis*) in Denmark from 2017 to 2021. Predictions are shown for models trained with observation data corrected for temporal and spatial bias, for models using AgERA5 weather data and Global Forecast System data. Values of point biserial correlation coefficient (*r*) are provided, measuring the association between predicted values and the dates of actual observations. All values are statistically significant (*α* = .001).

Model evaluation in selected regions also demonstrated high predictive performance and sensible predictions. For the Japanese beetle in northern Italy, the predicted values exhibited a strong correlation with the timing of observations, with a correlation coefficient of .8 or higher (figure 3, supplemental figure S8). For the winter chanterelle in Denmark, the correlations between predictions and observations were lower but remained strong with *r* values of .7 or higher (figure 4, supplemental figure S9).

**Figure 5.**
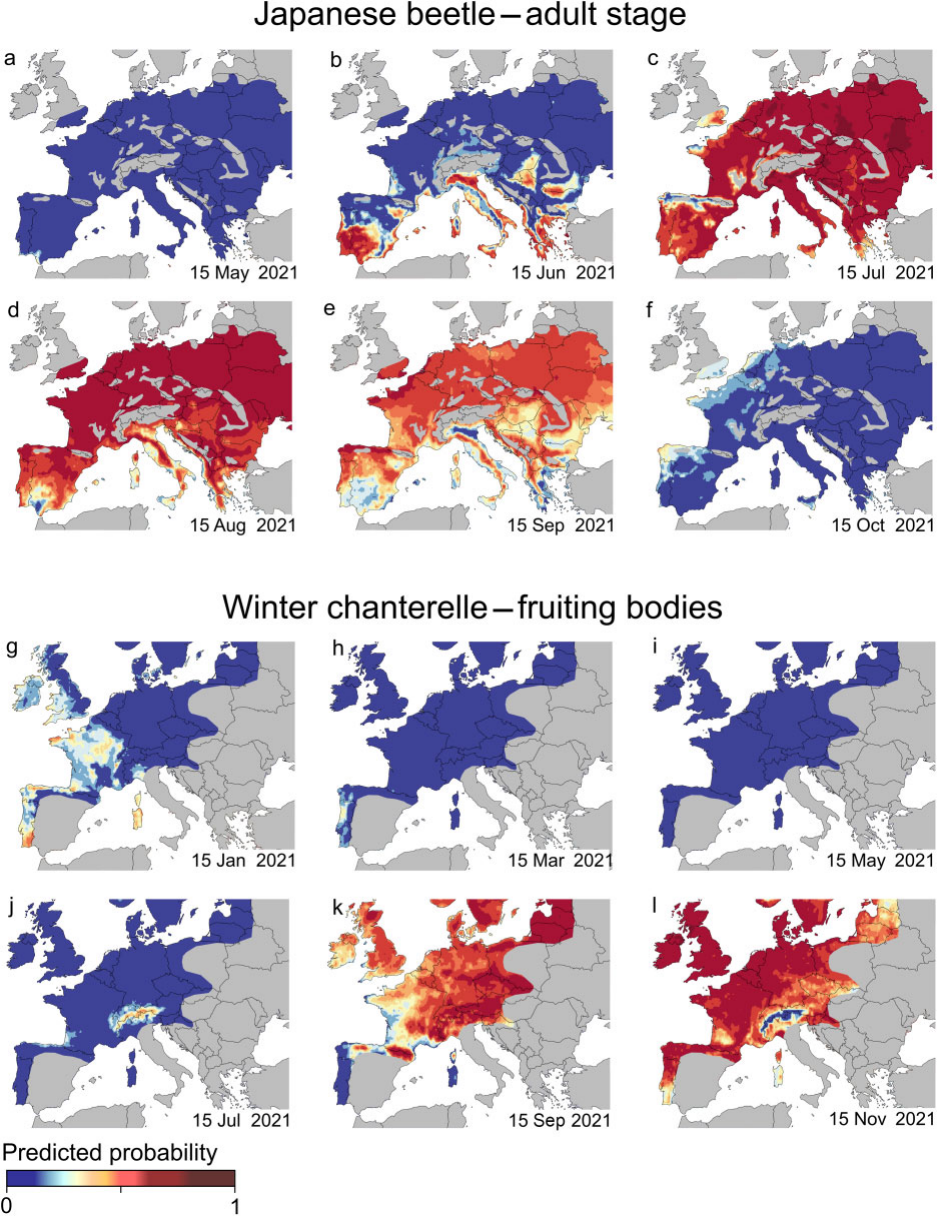
Predictions of the occurrence of adult Japanese beetles (*Popillia japonica*) (a–f) and of fruiting bodies of the winter chanterelle (*Craterellus tubaeformis*; g–l) across Europe on selected days of 2021. The predictions are based on models trained on observation data corrected for spatial and temporal bias and AgERA5 weather data. The areas in grey are expected to be unsuitable for the Japanese beetle (a–f) or are outside the distribution range of observation records of the winter chanterelle (g–l).

Maps of predictions for the Japanese beetle across Europe in 2021 show that adult beetles emerge earlier in southern regions (southern Iberia, southern France, southern Italy, and Greece), followed by most low-altitude regions in central and eastern Europe and later by the northern Iberian Peninsula, northern France, southern England, and some higher-altitude regions (figure 5a–5f, supplemental video V1). For the winter chanterelle, the early days of the year show moderate probabilities of fruiting bodies occurrence in southernmost regions (e.g., Portugal and Sardinia). The predicted values then drop across Europe before increasing around mid-July in the Alps, followed by most of Northern and Eastern Europe by mid-September, and expanding to southern regions thereafter (figure 5g–5l, supplemental video V2).

## Future prospects

We presented a methodological approach that allows predicting the timing of ecological events over wide geographical areas using opportunistic observation data, such as the data typically gathered from citizen science initiatives. The approach is theoretically grounded and was exemplified in the prediction of the emergence of adult Japanese beetles, an invasive species, and the availability of winter chanterelle fruiting bodies, an edible mushroom, across North America and Europe.

The approach demonstrated good predictive performance and strong agreement with observed patterns for both ecological phenomena. On the basis of the values of AUC—measuring the agreement between predictions and record labels for years left out of model training—the models for the Japanese beetle appear more robust than those of the winter chanterelle. However, the lower (but still good) AUC values achieved for the fruiting bodies of the winter chanterelle may be partly attributable to its longer season of suitable conditions, which extends up to approximately 5 months, compared with the 2.5–3 months for the adult stage of the Japanese beetle (EFSA 2019). This longer season results in the generation of a higher number of temporal pseudoabsences during periods that are environmentally suitable, thereby increasing the misclassification of these records in the evaluation data sets (Philips et al. 2009). Relevantly, the predictions were still robust when made for new areas—that is, under spatial transferability, a gold benchmark for spatial prediction in ecology (Roberts et al. 2017). This approach could be used to determine the optimal timing for surveillance efforts, particularly in the case of the Japanese beetle, a species that has not yet become established in most of Europe.

Although spatial and temporal biases in event observation data are not central to our modeling approach, they are a major source of contention in the development of predictions and estimates in temporal ecological research (Isaac and Pocock 2015). To address these biases, we proposed and tested a set of procedures on the basis of the patterns observed for a benchmark taxonomic group, which is believed to represent observer bias rather than taxon-specific phenology variation. Although we have demonstrated these procedures using *Pinus* spp. as a benchmark group, it is worth emphasizing that our methodology can seamlessly accommodate other taxonomic groups. For example, in regions outside the primarily northern hemisphere distribution of *Pinus* spp., researchers could use other taxonomic groups that better align with the characteristics of their study areas.

Crucially, despite the potential benefits of employing the benchmark taxa approach to address temporal biases, our results show that models accounting for these biases did not differ meaningfully in their predictive performance from those that did not account for them. This is likely because the models estimate the probability of a set of conditions being within relative phenological niches, rather than temporal trends per se. In other words, the models estimate the suitability of conditions on the basis of sampling data that does not need to be collected systematically across time and space. Instead, the representativeness of the data emerges from the joint sampling of conditions across regions and time periods. Therefore, although observational data may be biased and sparse in parts of its range, the combined use of all available observation records, representing suitable conditions, may allow for sampling most of the phenological niche.

Despite its demonstrated capability, there are several opportunities for future improvement of this approach. For instance, future work could explore general issues related to data-driven modeling, such as exploring additional predictive algorithms or different values in their parameterization. In addition, certain design choices could be further explored and optimized, such as the number of pseudoabsences to be extracted per observation record or the procedures used to translate environmental drivers into temporally discrete predictors. The expansion of our framework may also be necessary for more complex phenomena, such as those with temporal dependencies or interactions between events. Specifically, recursive fitting of models—that is, fitting the models with predictions for past periods could allow accounting for the temporal dependencies of specific phenological responses (Staggemeier et al. 2020). Similarly, predictions of interacting events could be performed and jointly modeled (Schermer et al. 2020). In addition, given the rapid pace of environmental change, it also seems essential to continuously calibrate and update these models using the most recent observation data through iterative modeling (Dietze et al. 2018). Given the emergence of novel environmental combinations in regions where the phenomena are observed, the lack of sampling of phenological responses under those settings may cause the models to fail. Therefore, continuous calibration using the most recent data is crucial to ensure that the models remain relevant and accurate over time.

## Conclusions

Our methodological approach allows for obtaining informative predictions of the timing of ecological events over wide geographical areas derived from abundant free and open records. The potential applications are vast, particularly considering the growing volumes of opportunistic observation data that are now available from various citizen science platforms. Further development and application of this approach is likely to make significant contributions to management-related activities such as ecological risk assessment, natural resource management, and conservation planning.

## Supplementary Material

biae041_Supplemental_Files

## Data Availability

The spatial weather data used for this work are publicly available online from Copernicus Climate Data Store and National Centers for Environmental Prediction. The event observation data are available from GBIF: https://doi.org/10.15468/dl.n2agbd, https://doi.org/10.15468/dl.cc6bbn, https://doi.org/10.15468/dl.jeq9wa, and https://mushroomobserver.org. The postprocessed data and R code are publicly available on Zenodo (https://zenodo.org/records/11124605).

## Author Biography

César Capinha (cesarcapinha@edu.ulisboa.pt) is affiliated with the Centre of Geographical Studies at the Institute of Geography and Spatial Planning of the University of Lisbon, in Lisbon, Portugal and the Associate Laboratory Terra in Lisbon, Portugal. Ana Ceia-Hasse (ana.ceia.hasse@cibio.up.pt) is affiliated with BIOPOLIS, CIBIO, and the InBIO Associate Laboratory, at the University of Porto, in Porto, Portugal and the University of Lisbon, in Lisbon Portugal. Sergio de-Miguel (sergio.demiguel@udl.cat) is affiliated with the Department of Agricultural and Forest Sciences and Engineering at the University of Lleida, in Lleida, Spain, and with the Forest Science and Technology Centre of Catalonia, in Solsona, Spain. Carlos Vila-Viçosa (cvv@cibio.up.pt) is affiliated with BIOPOLIS, CIBIO, and the InBIO Associate Laboratory and with the Museu de História Natural e da Ciência, at the University of Porto, in Porto, Portugal. Miguel Porto (mpbertolo@gmail.com) is affiliated with BIOPOLIS, CIBIO, and the InBIO Associate Laboratory, at the University of Porto, in Porto, and the University of Lisbon, in Lisbon, and with the Mértola Biological Station, in Mértola, Portugal. Ivan Jarić (ivan.jaric@hbu.cas.cz) is affiliated with Université Paris-Saclay, CNRS, AgroParisTech, Ecologie Systématique Evolution in Paris, France, and with the Biology Centre of the Czech Academy of Sciences, Institute of Hydrobiology, České Budějovice, Czech Republic. Patrícia Tiago (patricia.tiago@gmail.com) is affiliated with the Centre for Ecology, Evolution, and Environmental Changes & CHANGE--Global Change and Sustainability Institute, at Faculty of Sciences, University of Lisbon, in Lisbon, Portugal. Néstor Fernández (nestor.fernandez@idiv.de) and Jose Valdez (jose.valdez@idiv.de) are affiliated with the German Centre for Integrative Biodiversity Research (iDiv) Halle-Jena-Leipzig, in Leipzig, Germany and at Institute of Biology from the Martin Luther University Halle-Wittenberg, in Halle, Germany. Ian McCallum (mccallum@iiasa.ac.at) is affiliated with the International Institute for Applied Systems Analysis, in Laxenburg, Austria. Henrique Miguel Pereira (hpereira@idiv.de) is affiliated with the German Centre for Integrative Biodiversity Research (iDiv) Halle-Jena-Leipzig, in Leipzig, Germany and with the Institute of Biology from the Martin Luther University Halle-Wittenberg, in Halle, Germany, and with BIOPOLIS and CIBIO, in Porto, Portugal.
