# Supplementary material for: Using citizen science data for predicting the timing of ecological phenomena across regions
Source: Bioscience. 2024 Jul 9;74(6):383–92. doi: 10.1093/biosci/biae041 (PMC11266983; doi:10.1093/biosci/biae041)
Supplement: biae041_Supplemental_Files [file biae041_supplemental_files.zip › Capinha_et_al_BioScience_Supporting Information_R1.docx]

**Supplementary Material for: Predicting the timing of ecological phenomena across regions using citizen science data.**

César Capinha^a,b*^ PhD, Ana Ceia-Hasse^a,b^ PhD, Sergio de-Miguel^c,d^ PhD, Carlos Vila-Viçosa^e,f,g^ PhD, Miguel Porto^e,h,i,j^ PhD, Ivan Jarić^k,l^ PhD, Patricia Tiago^m^ PhD, Néstor Fernández PhD, Jose Valdez^n^ PhD, Ian McCallum^o^ PhD, Henrique Miguel Pereira^e,n,p^ PhD.

a. Centre of Geographical Studies, Institute of Geography and Spatial Planning, University of Lisbon, Rua Branca Edmée Marques, 1600-276 Lisboa, Portugal

b. Associate Laboratory Terra, Portugal

c. Department of Agricultural and Forest Sciences and Engineering, University of Lleida, Av. Alcalde Rovira Roure 191, E-25198 Lleida, Spain

d. Joint Research Unit CTFC – AGROTECNIO – CERCA, Ctra. Sant Llorenç de Morunys km 2, 25280 Solsona, Spain

e. BIOPOLIS Program in Genomics, Biodiversity and Land Planning, CIBIO, Campus de Vairão, 4485-661 Vairão, Portugal

f. MHNC-UP - Museu de História Natural e da Ciência da Universidade do Porto. Praça Gomes Teixeira, Sala 2.28/2.30, 4099-002 Porto, Portugal

g. Biology Department, Faculty of Sciences, University of Porto, Rua do Campo Alegre, s/n, 4169-007 Porto, Portugal

h. CIBIO, Centro de Investigação em Biodiversidade e Recursos Genéticos, InBIO Laboratório Associado, Campus de Vairão, Universidade do Porto, 4485-661 Vairão, Portugal

i. CIBIO, Centro de Investigação em Biodiversidade e Recursos Genéticos, InBIO Laboratório Associado, Instituto Superior de Agronomia, Universidade de Lisboa, 1349-017 Lisboa, Portugal

j. EBM, Estação Biológica de Mértola, 7750-329 Mértola, Portugal

k. Université Paris-Saclay, CNRS, AgroParisTech, Ecologie Systématique Evolution, Gif-sur-Yvette, France

l. Biology Centre of the Czech Academy of Sciences, Institute of Hydrobiology, České Budějovice, Czech Republic

m. cE3c, Centre for Ecology, Evolution and Environmental Changes, Faculdade de Ciências da Universidade de Lisboa, 1749-016 Lisboa, Portugal

n. German Centre for Integrative Biodiversity Research (iDiv) Halle-Jena-Leipzig

o. International Institute for Applied Systems Analysis, Schlossplatz 1, A-2361 Laxenburg, Austria

p. Institute of Biology, Martin Luther University Halle-Wittenberg, Am Kirchtor 1, 06108 Halle (Saale), Germany

***Corresponding author:** César Capinha | @: cesarcapinha@campus.ul.pt

**Supplementary Text.**

*Detailed description of rationale and procedures used to address temporal recording bias.*

To address temporal biases in our data, we employed a benchmark taxonomic group, *Pinus* spp. (i.e., pines), which we expect to experience variability in record availability mainly due to variation in observation effort rather than changes in the taxa phenology itself. Pines display limited visual changes throughout the year, with their evergreen foliage and discreet reproductive structures, making them a suitable choice for our benchmark group. Thus, we can assume that the temporal variation in the number of observation records for this taxon is mainly driven by extrinsic factors, such as weather conditions, day of the week, and time of the year, that affect biodiversity recording activity (Di Cecco et al. 2021).

We downloaded all observation records of *Pinus* spp. from GBIF made between 2015 and 2021, which had the full date of observation (i.e., day, month, and year) and geographic coordinates with a spatial precision greater than 0.1 decimal degrees. As for the Japanese beetle (*Popillia japonica*) and the winter chanterelle (*Craterellus tubaeformis*), we excluded records indicating the first day of the month and an observation time equal to '00:00:00'. Additionally, we accounted for multiple records resulting from a single recording session of the same specimen by considering all records having the same date within a 100 m radius from each other as a single record. We also identified areas of 250 × 250 km that were upper outliers in record numbers and downsampled the records in these regions by randomly selecting a number equal to the outlier threshold. Finally, to match with the distribution of records for *Craterellus tubaeformis* and *Popillia japonica* (Figs. S2 and S3), we only kept *Pinus* spp. records located in the northern hemisphere. In total, 87,691 *Pinus* spp. observation records were retained (Fig. S1).

Next, we generated an equal number of *Pinus* spp. observations having the same geographical coordinates, but with dates generated at random within the years (represented in the observation data (i.e., 2015 to 2021). This allowed us to obtain a distribution of records that would be expected if observations were made randomly over time. For both types of records (i.e., observations and randomly generated dates), we then extracted the day of the week, month, average temperature of the day, total precipitation of the day, and average wind speed of the day.

To ensure there were no collinearity issues among the predictors, we calculated the Variance Inflation Factor (VIF) using the car package (Fox and Weisberg 2019). Results showed no predictors surpassing problematic collinearity thresholds (i.e., all values of squared scaled generalised VIF ≤ 1.4). We then used a generalised linear model (GLM) with a binomial error distribution, to relate the two classes of records to the calendar and weather predictors. The fitted model returned sensible results, supporting an adequate capturing of temporal recording bias (see Results section in the main text).

We then applied the model to predict the level of sampling effort for the conditions associated with each record of the Japanese beetle and winter chanterelle. The values predicted represent the propensity for having more records simply because conditions are more favourable to observers (i.e., preferred days of the week, months, and weather conditions). We then accounted for this bias in the data sets of the Japanese beetle and the winter chanterelle using inverse probability weighting (Mansournia et al. 2016). Specifically, we built a second data set of observation records for each event, where the probability of each original observation being included was inversely proportional to the level of observation effort predicted. This corresponded to randomly selecting with replacement, the same number of total records, where the probability of each record being selected was defined as 1 minus the probability predicted by the model. In other words, records made under conditions less favourable for observers had greater chances of being selected, and vice versa.

**Supplementary References.**

Di Cecco, G. J., et al. (2021). Observing the observers: how participants contribute data to iNaturalist and implications for biodiversity science. BioScience, 71(11), 1179-1188.

Fox J, Weisberg S. 2019. An R companion to applied regression. Sage Publications.

Mansournia, M. A., & Altman, D. G. (2016). Inverse probability weighting. BMJ, 352, i189.

| **Table S1.** List of the 67 features used to characterize temporal environmental conditions for observation and temporal pseudo-absence records. ‘T’ stands for the date of the observation records and the associated numbers represent preceding days. | | | | | | | | | | | | | |
| --- | --- | --- | --- | --- | --- | --- | --- | --- | --- | --- | --- | --- | --- |
| **Geography** |  |  | **Mean temperature** |  | **Minimum temperature** |  | **Maximum temperature** |  | **Precipitation** |  | **Snow depth** |  | **Wind speed** |
| Latitude |  |  | Mean T -1 to T -2 |  | Mean T -1 to T -7 |  | Mean T -1 to T -7 |  | Sum T -1 to T -2 |  | Mean T -1 to T -5 |  | Mean T -1 to T -3 |
| Longitude |  |  | Mean T -1 to T -5 |  | Mean T -8 to T -14 |  | Mean T -8 to T -14 |  | Sum T -1 to T -4 |  | Mean T -1 to T -15 |  | Mean T -1 to T -7 |
|  |  |  | Mean T -6 to T -10 |  | Mean T -15 to T -21 |  | Mean T -15 to T -21 |  | Sum T -5 to T -8 |  | Mean T -16 to T -30 |  | Mean T -8 to T -14 |
|  |  |  | Mean T -11 to T -15 |  |  |  |  |  | Sum T -9 to T -12 |  |  |  |  |
|  |  |  | Mean T -16 to T -20 |  |  |  |  |  | Sum T -13 to T -16 |  |  |  |  |
|  |  |  | Mean T -21 to T -30 |  |  |  |  |  | Sum T -1 to T -8 |  |  |  |  |
|  |  |  | Mean T -31 to T -40 |  |  |  |  |  | Sum T -9 to T -16 |  |  |  |  |
|  |  |  | Mean T -41 to T -50 |  |  |  |  |  | Sum T -17 to T -24 |  |  |  |  |
|  |  |  | Mean T -51 to T -60 |  |  |  |  |  | Sum T -25 to T -32 |  |  |  |  |
|  |  |  | Mean T0 to T -29 |  |  |  |  |  | Sum T -33 to T -47 |  |  |  |  |
|  |  |  | Mean T -30 to T -59 |  |  |  |  |  | Sum T -48 to T -62 |  |  |  |  |
|  |  |  | Mean T -60 to T -89 |  |  |  |  |  | Sum T0 to T -29 |  |  |  |  |
|  |  |  | Mean T -90 to T -119 |  |  |  |  |  | Sum T -30 to T -59 |  |  |  |  |
|  |  |  | Mean T -120 to T -149 |  |  |  |  |  | Sum T -60 to T -89 |  |  |  |  |
|  |  |  | Mean T -150 to T -179 |  |  |  |  |  | Sum T -90 to T -119 |  |  |  |  |
|  |  |  | Mean T -274 to T -364 |  |  |  |  |  | Sum T -120 to T -149 |  |  |  |  |
|  |  |  | Mean T -182 to T -273 |  |  |  |  |  | Sum T -150 to T -179 |  |  |  |  |
|  |  |  | Mean T -182 to T -364 |  |  |  |  |  | Sum T -180 to T -209 |  |  |  |  |
|  |  |  | Mean T0 to T -365 |  |  |  |  |  | Sum T -274 to T -364 |  |  |  |  |
|  |  |  | Growing degree days since 1st Julian day (baseline 0ºC) |  |  |  |  |  | Sum T -182 to T -273 |  |  |  |  |
|  |  |  | Growing degree days since 1st Julian day (baseline 7ºC) |  |  |  |  |  | Sum T -182 to T -364 |  |  |  |  |
|  |  |  | Growing degree days since 1st Julian day (baseline 15ºC) |  |  |  |  |  | Sum T0 to T -365 |  |  |  |  |
|  |  |  | Growing degree days of past 90 days (baseline 2ºC) |  |  |  |  |  |  |  |  |  |  |
|  |  |  | Growing degree days of past 60 days (baseline 2ºC) |  |  |  |  |  |  |  |  |  |  |
|  |  |  | Growing degree days of past 30 days (baseline 2ºC) |  |  |  |  |  |  |  |  |  |  |
|  |  |  | Growing degree days of past 30 to 15 days (baseline 2ºC) |  |  |  |  |  |  |  |  |  |  |
|  |  |  | Growing degree days of past 15 days (baseline 2ºC) |  |  |  |  |  |  |  |  |  |  |
|  |  |  | Growing degree days of past 7 days (baseline 2ºC) |  |  |  |  |  |  |  |  |  |  |
|  |  |  | Cold accumulation since 1st Julian day (baseline 5ºC) |  |  |  |  |  |  |  |  |  |  |
|  |  |  | Cold accumulation since 1st Julian day (baseline 10ºC) |  |  |  |  |  |  |  |  |  |  |
|  |  |  | Cold accumulation of past 30 days (baseline 5ºC) |  |  |  |  |  |  |  |  |  |  |

**Table S2.** Results of a generalized linear model with binomial error distribution, relating the presence or absence of records of the benchmark taxonomic group (*Pinus* spp.) and predictors representing calendar- and weather-related conditions for matching days.

|  | Estimate | Std. Error | P | Sig. |
| --- | --- | --- | --- | --- |
| (Intercept) | -7.322 | 0.213 | < 2e-16 | *** |
| Monday | -0.047 | 0.019 | 0.012455 | * |
| Saturday | 0.348 | 0.018 | < 2e-16 | *** |
| Sunday | 0.278 | 0.018 | < 2e-16 | *** |
| Thursday | -0.02 | 0.019 | 0.287569 |  |
| Tuesday | -0.055 | 0.019 | 0.003422 | ** |
| Wednesday | 0.023 | 0.019 | 0.214283 |  |
| August | 0.074 | 0.023 | 0.001493 | ** |
| December | -0.69 | 0.027 | < 2e-16 | *** |
| February | -0.614 | 0.027 | < 2e-16 | *** |
| January | -0.758 | 0.028 | < 2e-16 | *** |
| July | 0.218 | 0.023 | < 2e-16 | *** |
| June | 0.026 | 0.023 | 0.26644 |  |
| March | -0.412 | 0.025 | < 2e-16 | *** |
| May | 0.075 | 0.023 | 0.000927 | *** |
| November | -0.511 | 0.026 | < 2e-16 | *** |
| October | -0.203 | 0.023 | < 2e-16 | *** |
| September | -0.172 | 0.024 | 3.55E-13 | *** |
| Mean temperature | 0.026 | 0.001 | < 2e-16 | *** |
| Total precipitation | -0.024 | 0.001 | < 2e-16 | *** |
| Mean wind speed | -0.013 | 0.003 | 6.29E-05 | *** |

Significance codes: ‘***’ 0.001; ‘**’ 0.01; ‘*’ 0.05

**Supplementary Figures**


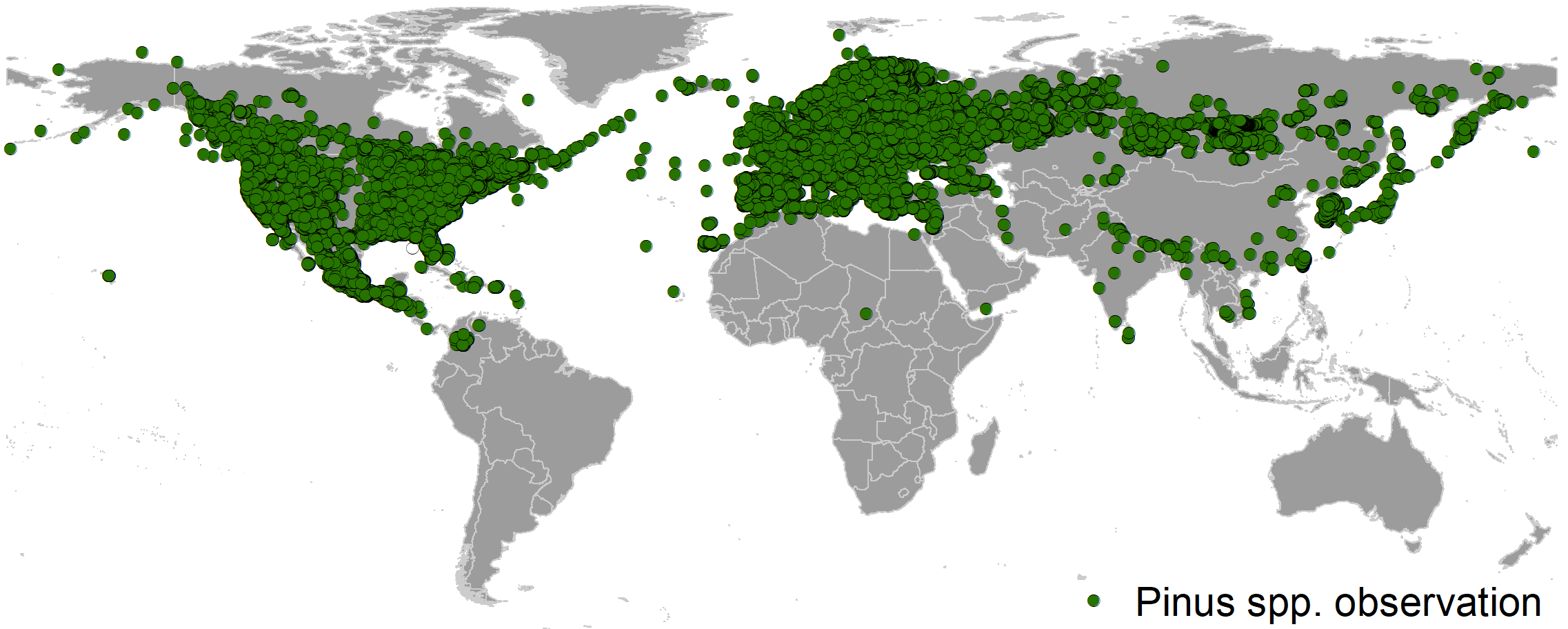


**Figure S1.** Distribution of observation records of *Pinus* spp. used to identify patterns of temporal bias in recording effort.

**
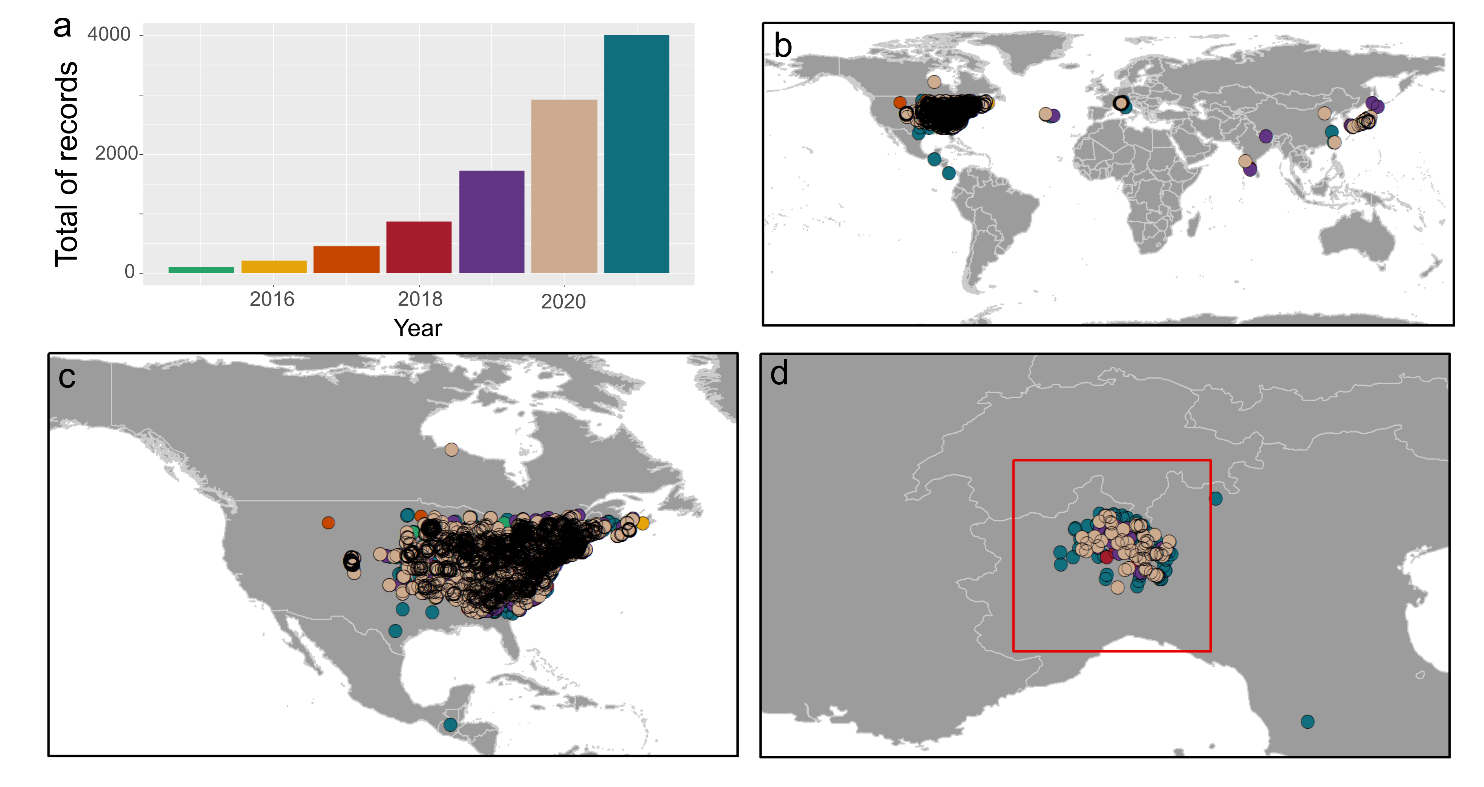
**

**Figure S2.** Temporal (a) and spatial (b-d) distribution of observation records of the Japanese beetle (*Popillia japonica*), after accounting for geographic overrepresentation, i.e., used for modelling. The red square in panel d delimits the observation records in Italy that were used for model validation in this region.

**
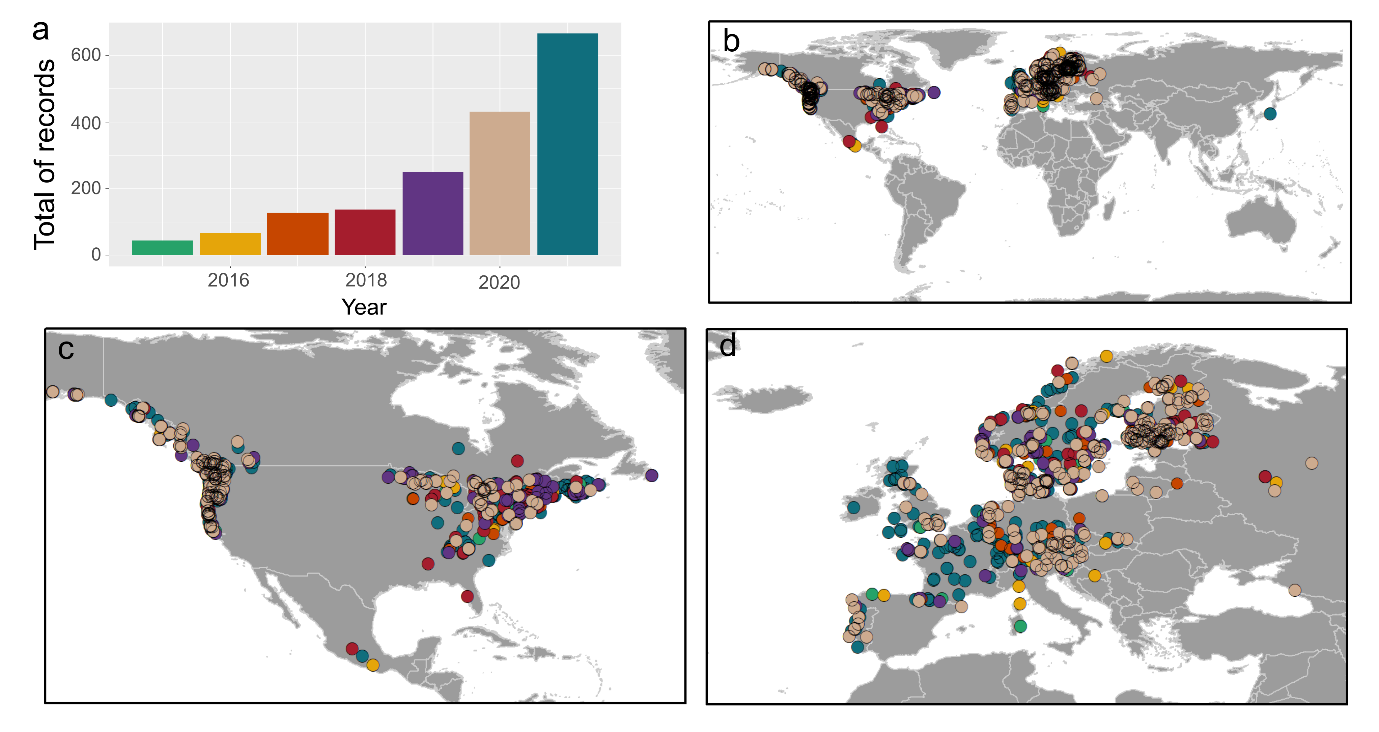
**

**Figure S3.** Temporal (a) and spatial (b-d) distribution of observation records of the winter chanterelle (*Craterellus tubaeformis*), after accounting for geographic overrepresentation, i.e., used for modelling.


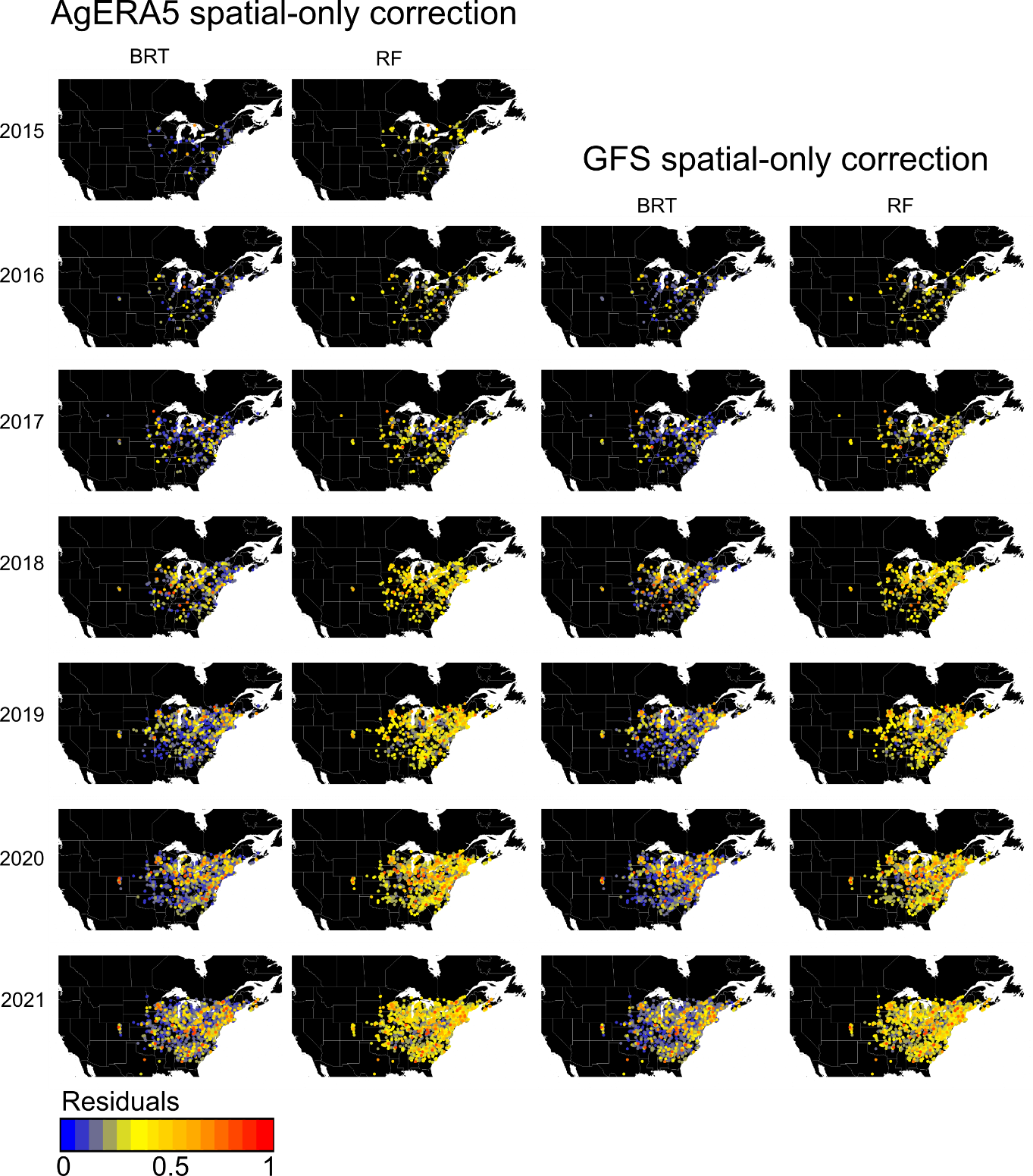


**Figure S4.** Residual values of models for the Japanese beetle (*Popillia japonica*) trained on observation data without temporal bias correction.


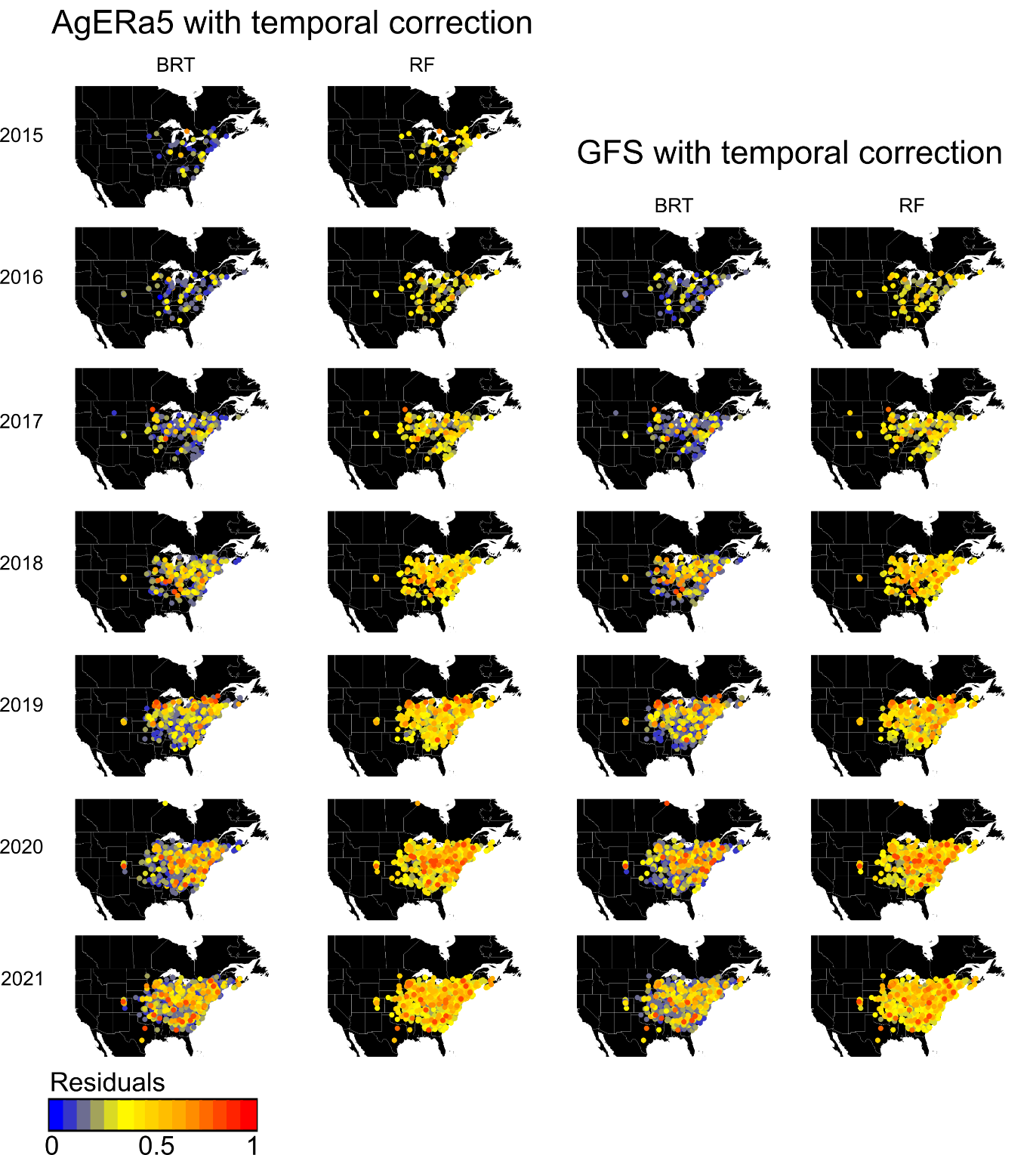


**Figure S5**. Residual values of models for the Japanese beetle (*Popillia japonica*) trained on observation data with temporal bias correction.


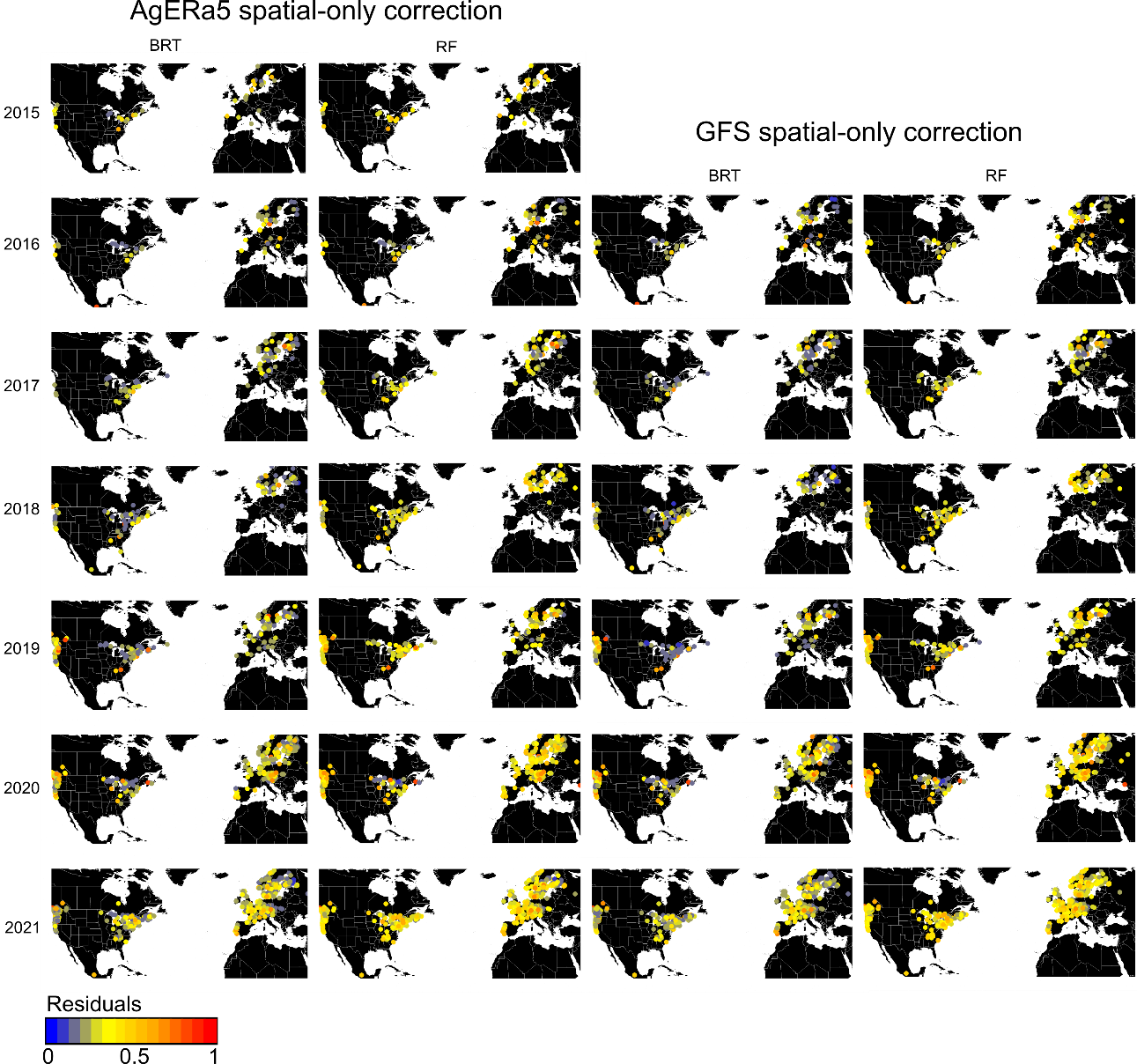


**Figure S6.** Residual values of models for the winter chanterelle (*Craterellus tubaeformis*) trained on observation data without temporal bias correction.


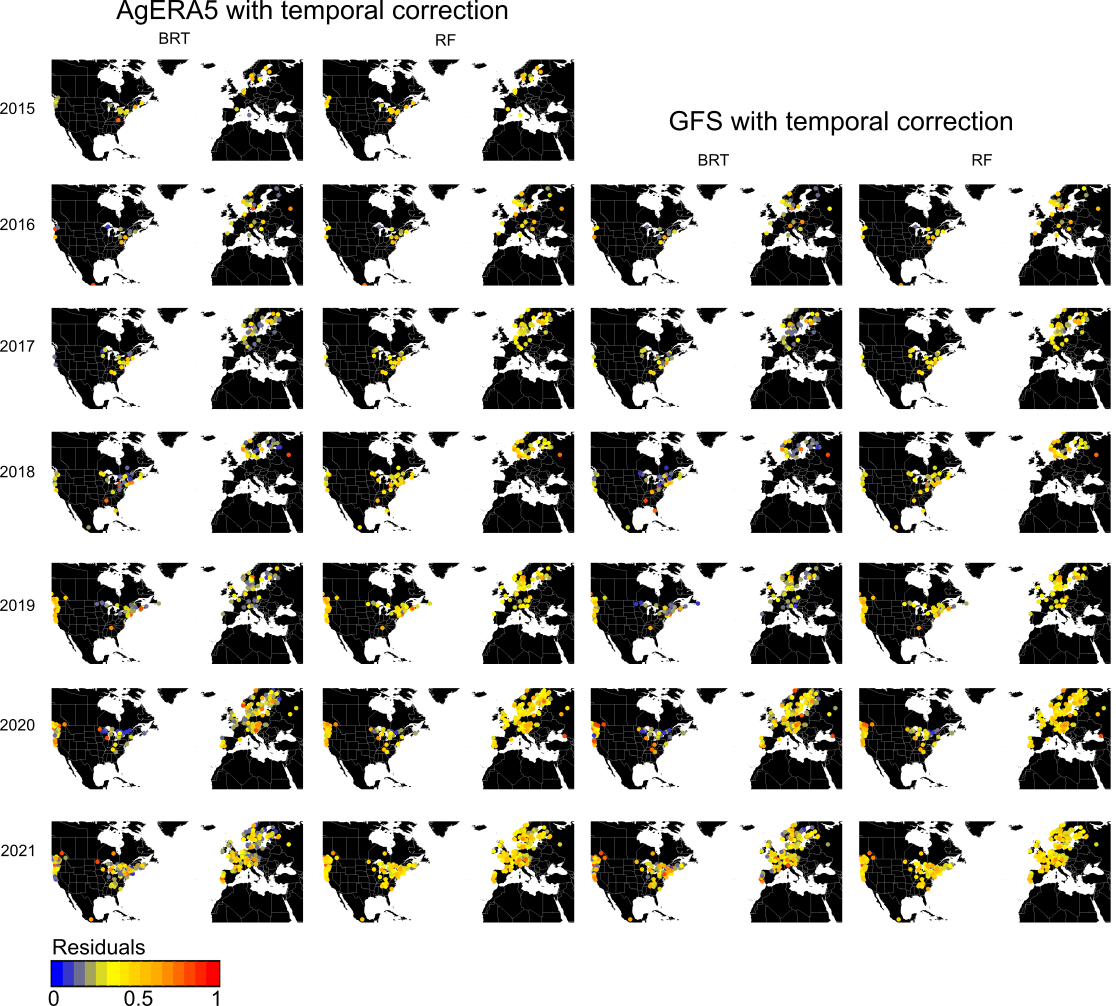


**Figure S7.** Residuals of models for the winter chanterelle (*Craterellus tubaeformis*) trained on observation data with temporal bias correction.


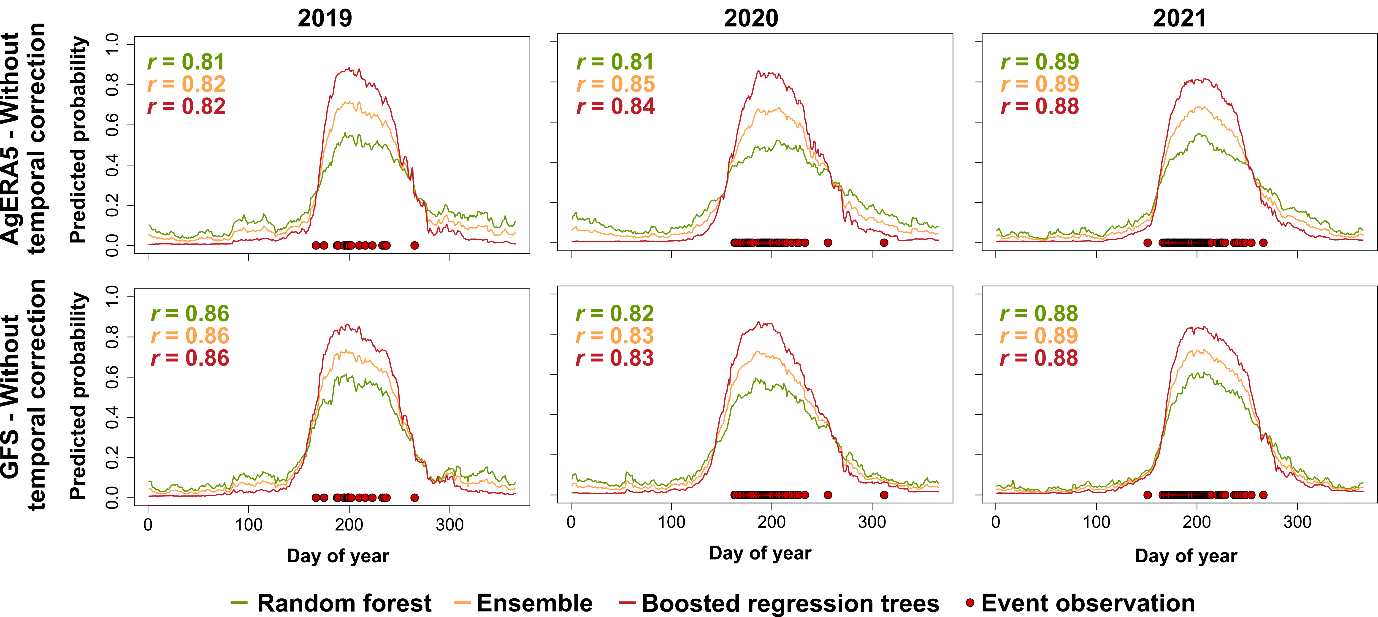


**Figure S8.** Continuous predictions of the timing of occurrence of adult Japanese beetles (*Popillia japonica*) in northwest Italy from 2019 to 2021. Predictions are shown for models trained with observation data corrected for spatial bias only, for models using AgERA5 weather data and Global Forecast System data (GFS). Values of Point Biserial Correlation coefficient (*r*) are provided, measuring the association between predicted values and the dates of actual observations. All values are statistically significant (α=0.001).

**
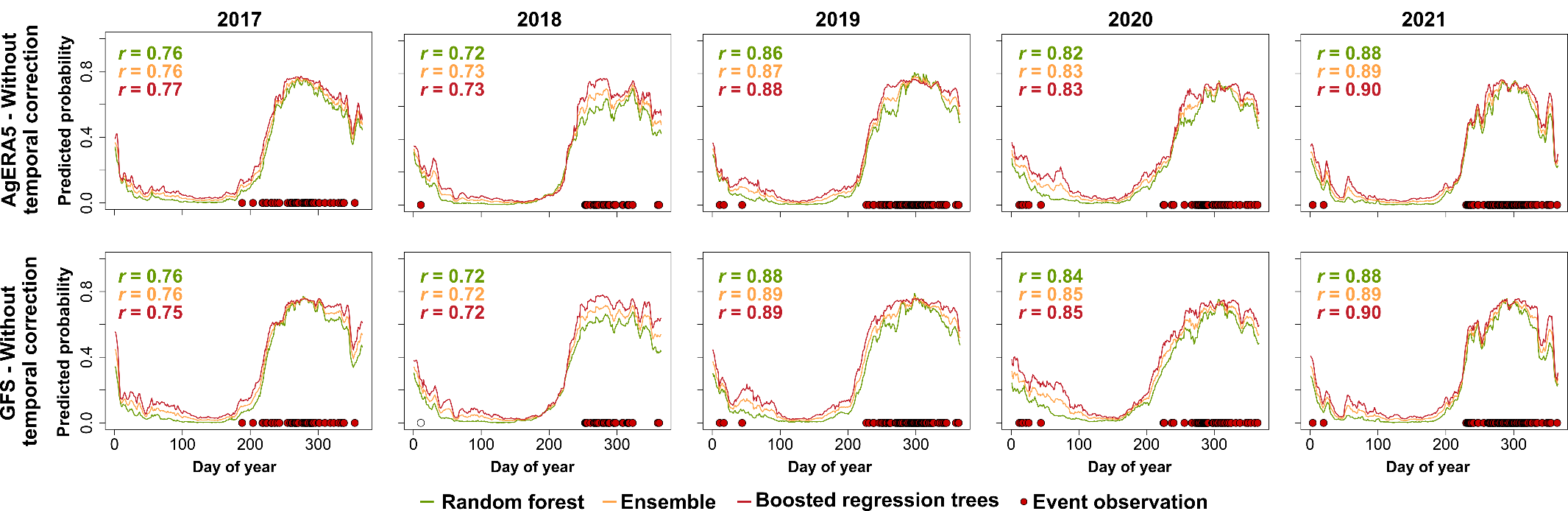
Figure S9.** Continuous predictions of the timing of occurrence of fruiting bodies of the winter chanterelle (*Craterellus tubaeformis*) in Denmark from 2017 to 2021. Predictions are shown for models trained with observation data corrected for spatial bias only, for models using AgERA5 weather data and Global Forecast System data (GFS). Values of Point Biserial Correlation coefficient (*r*) are provided, measuring the association between predicted values and the dates of actual observations. All values are statistically significant (α=0.001).
